# Algorithm of Stability-Analysis-Based Feature Selection for NIR Calibration Transfer

**DOI:** 10.3390/s22041659

**Published:** 2022-02-20

**Authors:** Zheyu Zhang, Yaoxiang Li, Chunxu Li, Zichun Wang, Ya Chen

**Affiliations:** College of Engineering and Technology, Northeast Forestry University, Harbin 150040, China; zheyuzhang@nefu.edu.cn (Z.Z.); chunxuli.lcx@gmail.com (C.L.); wangzichun@nefu.edu.cn (Z.W.); ya_chen@nefu.edu.cn (Y.C.)

**Keywords:** near-infrared spectroscopy, calibration transfer, feature selection, stability analysis

## Abstract

For conventional near-infrared spectroscopy (NIR) technology, even within the same sample, the NIR spectral signal can vary significantly with variation of spectrometers and the spectral collection environment. In order to improve the applicability and application of NIR prediction models, effective calibration transfer is essential. In this study, a stability-analysis-based feature selection algorithm (SAFS) for NIR calibration transfer is proposed, which is used to extract effective spectral band information with high stability between the master and slave instruments during the calibration transfer process. The stability of the spectrum bands shared between the master and slave instruments is used as the evaluation index, and the genetic algorithm was used to select suitable thresholds to filter out the spectral feature information suitable for calibration transfer. The proposed SAFS algorithm was applied to two near-infrared datasets of corn oil content and larch wood density. Simultaneously, its calibration transfer performances were compared with two classical feature selection methods. The effects of different preprocessing algorithms and calibration transfer algorithms were also assessed. The model with the feature variables selected by the SAFS obtained the best prediction. The SAFS algorithm can simplify the spectral data to be transferred and improve the transfer efficiency, and the universality of the SAFS allows it to be used to optimize calibration transfer in various situations. By combining different preprocessing and classic feature selection methods with this, the sensitivity of the correlation between spectral data and component information are improved significantly, as well as the effect of calibration transfer, which will be deeply developed.

## 1. Introduction

As one of the green and non-destructive testing methods, near-infrared spectroscopy has been used to analyze qualitative and quantitative data by constructing multivariate correction models. At present, it has been widely used in petrochemical, pharmaceutical, agriculture, forestry, and other fields [1,2,3,4]. However, the basic characteristics of the tested sample, the differences in the structure and principles between spectrometers, and the variations in the detected environment all have influenced on the reliability of the model, and can cause signal drift and absorption peak shape changes in the results of near-infrared detection, resulting in the disturbed accuracy of the model [5]. Hence, ow to obtain an accurate and stable multivariate calibration mathematical model has become a central issue.

In actual production, the problem of poor generality among spectral models has hindered the development of spectral analysis technology. There are two common reasons: one is that the samples to be tested have changed. Due to the difference in time or testing environment (temperature, humidity, noise), performance differences appear between the samples used for testing and analysis, at which time, direct analysis of existing samples with the original model is not sufficient to meet the practical needs [6]. The second is the different types of spectrometers or principles of measurement. The differences in processing technology and mechanical structure lead to the difficulty of using a common model for the same batch of instruments, at the same time, the differences in instruments also hinder the sharing of production experience or scientific research technology [7]. Calibration transfer is one of the effective methods to solve the above technical problem at present [8], which eliminates the time-consuming nature and workload of model reconstruction and has far-reaching implications for the application and development of NIR techniques.

In recent years, miniaturized near-infrared spectrometers have been favored by the majority of manufacturers and markets. With their flexibility and versatility, they are widely used in medical, industrial, agricultural, and other fields [9]. However, it is difficult to share data between different spectrometers due to the different manufacturing processes and specifications of various miniaturized spectrometers, which limits the development of NIR spectroscopy in the current big data context. At present, some researches have been devoted to the calibration transfer between small-scale near-infrared spectrometers and laboratory spectrometers [10], the calibration transfer between different small-scale near-infrared spectrometers [11], and the calibration transfer between different tested samples [12]. However, there are still few systematic studies in this field, and further exploration is necessary.

Traditional calibration transfer algorithms have been widely used, and are often used to compare and test the reliability of new algorithms. They can be divided into two categories according to their having or not having standard samples. Standardless samples analysis methods, such as orthogonal signal correction (OSC) [13], finite impulse response (FIR) [14], etc., have been widely applied to transform and reduce the dimension of the spectrum in the pretreatment stage, thereby enhancing the readability of the spectrum and reducing the influence of noise and other irrelevant factors on the spectrum. Standard samples analysis methods normally construct the conversion coefficient matrix between the master and slave instruments by standardizing the data, to achieve the data reconstruction of the slave instrument, including direct standardization (DS) [15], piecewise direct standardization (PDS) [16], slope–bias correction (SBC) [17], Shenk’s algorithm [18], etc. Recently, some novel algorithms—such as data-standardization-based spectral space transformation (SST) [19], factor-analysis-based canonical correlation analysis algorithm (CCA) [20], and mapping spectral data to low-dimensional space, based the spectral regression method [21], etc.—have been reported, which confirmed that these methods had better calibration transfer effects than traditional methods.

The traditional algorithm requires high similarity between transfer objects and a large number of labeled samples, which limits the transferability and application range of near-infrared technology. Therefore, a variety of calibration transfer algorithms have been proposed from different directions, such as non-linearity, machine learning, transfer learning, and standardless samples. Liu et al. [22] proposed an approach for calibration transfer, based on the alternating trilinear decomposition (ATLD) method combined the master and slave instruments spectrum data to form a three-way spectral matrix, and calculated the coefficients in the relative intensity to standardize the data. Liu et al. [23] proposed a linear model correction (LMC) method for calibration transfer without standard samples, using the constrained optimization method to standardize the coefficients of the models between the two instruments to achieve calibration transfer. Based on the principle of transfer learning, Yu et al. [24] had been achieved calibration transfer for different types of samples by using principal component analysis (PCA) combined with boosting extreme learning machine (ELM). Li et al. [25] used partial least squares to reduce the dimensionality of data, indicating that the prediction of constructed model was improved through transfer learning. Considering that there may be a nonlinear relationship between the instruments, Shan et al. [26] proposed a nonlinear calibration transfer method based on joint kernel subspace. According to the nonlinear characteristics between the master and slave instruments, the spectral data was reconstructed, and then the nuclear partial least quadratic method was used to establish the transfer model, through comparison, obtaining a similar result as the linear method.

However, the full bands or the characteristic bands of the master instrument are often used in the modeling of the above methods. Although the efficiency is improved in this way, the corresponding characteristic bands between the master and slave instruments are ignored. Therefore, many targeted feature selection methods have been proposed for calibration transfer [27,28,29] to improve efficiency and prediction accuracy. Xu et al. [30] proposed a correlation-analysis-based wavelength selection method (CAWS); it constructed the Pearson’s correlation coefficient matrix of the correlation between the master and slave instruments data, and determined the most suitable characteristic wavelength for calibration transfer according to the threshold value, finding that the improvement of prediction was obvious under full-spectrum conditions. Li et al. [31] proposed a new relative error analysis (REA) wavelength selection method based on relative errors, reducing redundant variables between delivery models, and calibration transfer of different fruit products was realized. Zhang et al. [32] combined screening wavelengths with consistent and stable signals (SWCSS) algorithm with traditional feature selection methods to further filter the feature wavelengths used for calibration transfer and improve the accuracy of the model. When using these methods to select features, only the relevant analysis is performed on the wavelength of the master and slave instruments, but the influence of the instability of the wavelength drift is not considered, and some of them are too complicated, which reduces the efficiency of calibration transfer.

Among the shortcomings of the above methods, we propose a stability-analysis-based feature selection algorithm (SAFS). According to the characteristics of the stability [33], the joint stabilities of the spectrum between the master and slave instruments are calculated by SAFS, the wavelengths with high stability are selected to construct the master model and calibration transfer for the slave model. Meanwhile, the genetic algorithm is used to determine the appropriate threshold to ensure a close relationship between the wavelength and the target component. The wavelength selected by the SAFS algorithm is not optimal for the construction of the master model, but the prediction accuracy and robustness of the slave model has been improved significantly, because it is an algorithm designed for calibration transfer specifically. The performance of the proposed SAFS algorithm was verified by using corn data, measured by different instruments, and larch wood data, involved in different moisture contents, and it was compared to those of other feature selection methods, under different pretreatment and calibration algorithm conditions.

## 2. Materials and Principles

### 2.1. Stability-Analysis-Based Feature Selection Method (SAFS)

In the partial least squares (PLS), the independent variable, *X*_*m*×*n*_, and the dependent variable, *Y*_*m*×1_, have the following linear relationship [34]:

(1) $$Y_{m\times 1}=X_{m\times n}\beta_{n\times 1}+E$$

For spectral data, the independent variable, *X*, is an *m × n* response matrix containing *m* samples and *n* spectral response variables; the dependent variable, *Y*, is the component information corresponding to *m* samples; *β* is a coefficient vector containing *n* regression coefficients; and *E* is the random error vector. The absolute value of *β* and *E* can reflect the contribution of each response variable to the built model, and the valid information variables of the spectrum are distinguished. According to this feature, we propose the SAFS algorithm. The specific process follows.

First, master and slave instruments samples are divided into correction set, validation set, and prediction set, according to the same ratio. Using the Monte Carlo method, the calibration sets *a* and *b* of the master and slave instruments are randomly sampled at a fixed ratio, *P*, and modeled separately. Monte Carlo sampling has been executed *K* times in total. The characteristic wavelengths can be measured by the stability index, and the absolute value of the stability of each wavelength point can be calculated. The definition of stability is as follows [35]:

(2) $$\left|c_{i}\right|=\left|\frac{\bar{a_{i}}\cdot \bar{b_{i}}}{\sqrt{\frac{\sum_{j=1}^{K}{\left(a_{ij}-\bar{a_{i}}\right)}^{2}}{K-1}}\cdot \sqrt{\frac{\sum_{j=1}^{K}\left(b_{ij}-\bar{b_{i}}\right)}{K-1}}}\right|$$

where *a_ij_* and *b_ij_* represent the regression coefficient measured by the *j*th Monte Carlo sampling at the *i*th response variable using the master and slave instruments, respectively. $\bar{a_{i}}$ and $\bar{b_{i}}$ represent the mean value of the regression coefficient measured at the *i*th response variable in the *K* times of Monte Carlo sampling using the master and slave instruments, respectively. *c_i_* is the ratio of the mean value to the standard deviation of the regression coefficient, which is used to measure stability.

**Figure 1:**
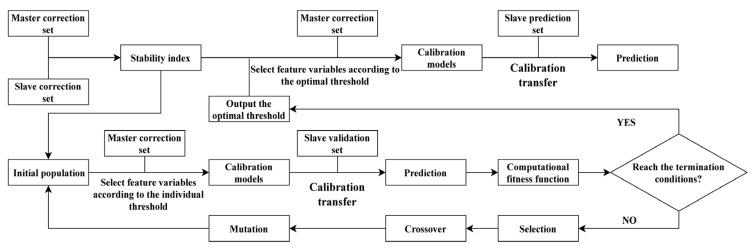
The framework of SAFS.

Determine the maximum value |c_i_|_max_ and minimum value |c_i_|_min_ of |c_i_| as the optimal value range of the stability threshold, T (T (|c_i_|_min_, |c_i_|_max_)). According to the property of stability, if the regression coefficient measured by the master and slave instruments had a high average value and a low standard deviation, the stability would be high, and the corresponding *i*th response variable would be important, and its modeling and prediction ability has been improved, so that all |c_i_| > T corresponding response variables were characteristic wavelengths. Using the characteristic wavelengths selected by SAFS, the transferred prediction set is substituted into the master model and assessed the prediction result. The optimization process of SAFS algorithm is shown in Figure 1.

For the stability calculated by each Monte Carlo sampling, the regression coefficient is calculated by using 10-fold cross-validation to eliminate the overfitting in the modeling process (Figure 1). The calibration transfer algorithm mainly includes three types, namely, the conversion matrix of the master–slave instrument spectrum data is established with sample standard; the spectrum data is converted from slave to the master instruments without sample standard; and the prediction result and component information are linearly calibrated. The SAFS algorithm can be used in combination with the above algorithms.

### 2.2. Determination of the Optimal Threshold of SAFS with Genetic Algorithm

In stability analysis, the selection of the optimal threshold value is crucial. If the threshold value is too large, some valid feature information may be eliminated, while some invalid information and noise may be introduced if the threshold value is too small. In this study, a genetic algorithm (GA) [36] was used for threshold optimization. The initial population number was set to 50, the chromosome binary code length was set to 20, the maximum evolutionary algebra was set to 100, the crossover probability was set to 0.8, and the mutation probability was set to 0.1. The upper and lower wavelengths of the threshold T correspond to the maximum and minimum values of stability |c_i_|_max_ and |c_i_|_min_. For the fitness function, the feature variables in the correction set of each population are screened according to the individual threshold, and the contiguous data blocks cross-validation is used to establish the PLS model for calibration transfer. Validation set for the slave instrument was then used for the above model prediction. The coefficient of determination (*R*^2^) and the root mean square error of the validation set (RMSEV) were calculated and the fitness function was obtained as follows:

(3) $$F=\frac{R^{2}}{1+\mathrm{RMSEV}}$$

It can be seen that a better prediction effect of the validation set is associated with larger *R*^2^ and fitness function value, *F*, and a smaller RMSEV, and the stability of the wavelengths shared by the selected master and slave instruments is higher. At this time, the threshold is considered to be the best. The roulette wheel selection copy operation, probability-based crossover and mutation operations are used for the generated population to generate a new population, and the best individuals in the past are retained in the new population; finally, the algorithm iterates to meet the final conditions or reach the maximum heredity algebra, output the optimal threshold T_best_.

## 3. Data Processing

In this study, two datasets of near-infrared spectroscopy are used to verify the proposed SAFS algorithm. The first spectroscopy dataset is public corn sample data, downloaded from http://www.software.eigenvector.com/Data/Corn/index.html (accessed on 27 January 2022). The second dataset is larch wood samples data, which were collected from Xinghuo Forest Farm (45°43′5.73″ N, 129°13′34.37″ E), Fangzheng County, Heilongjiang Province, China.

### 3.1. Corn Dataset

The corn dataset consists of 80 corn samples measured by 2 different near-infrared spectrometers, namely: mp5 and mp6. Each spectra spanned the wavelength range from 1100 nm to 2498 nm with digitization interval of 2 nm, composing of 700 data points. In addition, the spectral data in the database are all original spectra without any preprocessing. The spectral response variable includes the moisture, oil, protein, and starch content of each corn sample. In this study, designating mp5 as the master instrument and mp6 as the slave instrument, and the oil content of the corn sample was studied. More specifically, the slave instrument data is transferred to the master instrument, and the main model is created to predict the transmitted data.

### 3.2. Larch Wood Dataset

The larch samples were collected from the natural secondary forest farm of larch. Four plots on the sunny side and the shaded side were set up with a plot size of 20 m × 20 m. Additionally, three typical sample trees were selected from each plot. After each sample tree was felled, the portable chain saw was used to cut multiple wood discs continuously from bottom to top near the standard diameter at breast height (1.3 m at breast height). Brought it back to the laboratory and peeled it by hand, the wood strips of 2 cm × 2 cm × 4 cm were extracted from the wooden discs with a total of 181 larch wood samples. Each sample was labeled and recorded. The samples were placed in a ventilated and dry room-temperature (20 °C) environment for 4 weeks and their equilibrium moisture content was about 10%, then we determined the air-dried density of the wood samples according to the International Standard Organization (ISO) 13061-2: 2014 [37].

To avoid the effects caused by surface roughness, 80-mesh sandpaper was used to polish each side of the sample 5 times to make the surface roughness parameter Ra close to 12.5 μm. We controlled the temperature at 20 °C and set the moisture content to be 70%, 50%, 30%, and 10% in four groups, respectively. The air-dried wood samples were soaked in water for 20 days, and then dried in an oven, weighed, and we calculated the moisture content of the sample every 5–15 min after drying, until the moisture content of the sample was within the range of the specified variation group. When the specified moisture content value was reached, we measured the near-infrared spectrum data of the sample immediately. A portable spectrometer with a wavelength range of 350–2500 nm and composing of 2151 data points, ASD LabSpec^®^ Pro FR/A114260 was used to measure the spectrum. The fiber optic probe was used to scan once each at two different positions on the cross-section of the sample. Each scan time was about 1.5 s. The sample was continuously scanned 30 times during the set scan period. The average of the two measurements was taken as the original spectral data.

**Figure 2:**
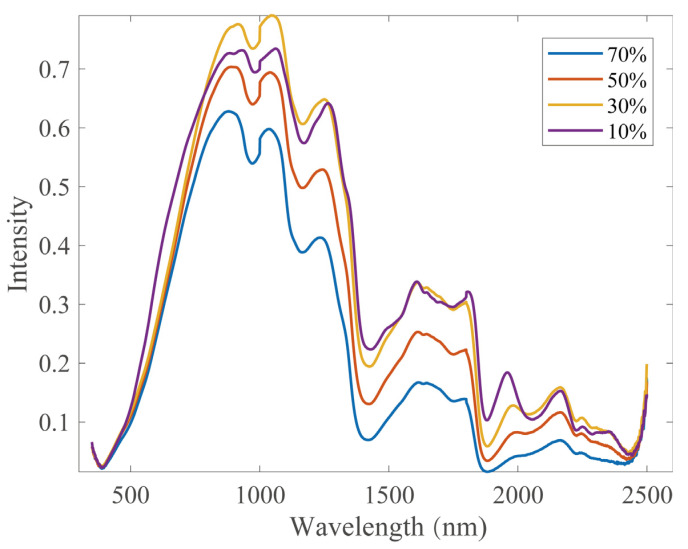
Near-infrared spectra for wood samples with different moisture content (70%, 50%, 30%, 10%).

It shows that the NIR spectral vary to some extent with the moisture content of the wood sample (Figure 2), such as baseline drift, drift of a small part of the absorption peak, and change in the absorption peak shape. However, the overall trend as well as the absorption peak of the spectral is pretty similar. The internal structure of the wood sample is varied with moisture content, which could cause variation of the spectrum. Additionally, the location and scanning angle of the probe could also cause variation of the spectrum. In this study, we used the wood NIR model with 10% moisture content as the calibration master model, and used spectral data for wood samples at other moisture content levels (70%, 50%, 30%) as the slave model data, and the calibration transfer was investigated in terms of the measuring environment.

### 3.3. Data Processing

For the corn dataset, we verified the optimized performance of various spectral preprocessing methods, and standard normal variate transformation (SNV) [38] obtained the predicted results (*R*^2^ = 0.6993, RMSE = 0.0980). Therefore, SNV was used as the spectral preprocessing method in this study. Additionally, SNV method is the same choice as Xu et al. [30] which can be used to evaluate the performance of the SAFS. For the larch wood dataset, the 21-point Savitzky–Golay smoothing algorithm was used to eliminate noise [39], the 1st derivative of the spectral data was used to baseline correction [40], and then we eliminated the influence of particle size and scattering on the spectrum of the sample surface by combining SNV. Specifically, the high leverage value combined with the residual Student’s *t*-test method [41] was used to screen the singular sample numbers of the four moisture content groups in the larch wood dataset. Four groups of outlier numbers were merged into one, and the sample data corresponding to the serial number of the four groups of data were removed. Finally, 13 samples of No. 6, 12, 20, 22, 27, 38, 39, 41, 44, 45, 47, 56, and 154 were eliminated, and a total of 168 samples of larch wood were obtained. The sample set partitioning, based on the joint x–y distances (SPXY) method [42], was used to divide the sample set into the correction set, validation set, and prediction set. Among them, the calibration set, validation set, and prediction set of corn samples each had 45, 15, and 20 samples, respectively; the calibration set, validation set, and prediction set of larch wood samples each had 95, 31, and 42 samples, respectively.

The optimal principal factor number for the calibration model was determined by principal component analysis (PCA). Four representative algorithms, PDS, SBC, SST, and OSC, were used in the calibration transfer. The SBC algorithm is a correction algorithm for the prediction result. PDS and SST algorithms are calibration transfer algorithms with standard samples which constructed a spectrum data conversion matrix between master and slave instruments. For the conversion matrix, the PDS algorithm constructs it through a moving window; the SST algorithm constructs it through singular decomposition to separate the noise and useful information. In this study, for PDS, we set the window size to 9. For SST, the one-by-one substitution method was used to calculate the number of SST factor of the validation model, and it was considered that the optimal number of SST factors occurred when RMSEV of validation model was the smallest. The OSC algorithm was a calibration transfer algorithm without standard samples.

In this study, the number of Monte Carlo iterations of the SAFS algorithm was set to 1000, and the sampling rate was set to 0.8. Four methods of the synergy interval PLS (SiPLS) [43], competitive adaptive reweighted sampling algorithm (CARS) [44], feature-free selection (“none”), and SAFS were used for wavelength selection. The calculations were run in MATLAB software, version R2017a (Mathwork, American).

## 4. Results and Discussion

### 4.1. Calibration Transfer of Corn Dataset

For the calibration transfer between different instruments, based on the corn dataset, the spectral wavelengths were selected by SAFS, CARS, and SiPLS methods, respectively. Additionally, then the calibration transfer results were compared and evaluated.

**Figure 3:**
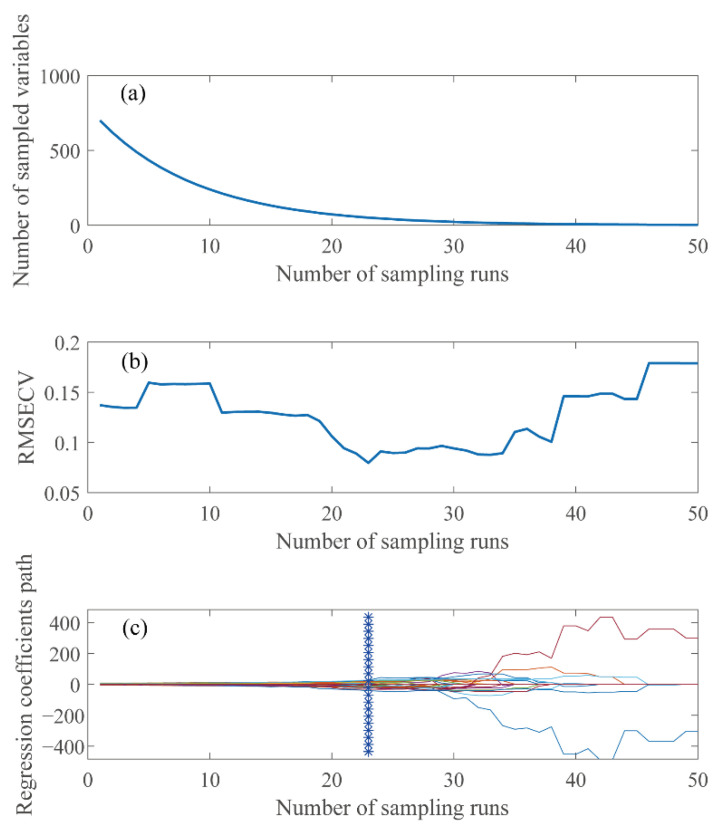
Feature selected by CARS method of the mp5 sample set, the trend graph of (**a**) number of sampled variables, (**b**) RMSECV, and (**c**) variable stability path changes with the subset of wavelength variables. Note: The asterisk line in the figure indicates the number of subsets corresponding to the smallest RMSECV.

When using CARS method, the Monte Carlo sampling was run 50 times with sampling rate of 0.8, the PLS model was constructed using 10-fold cross-validation, in which the maximum latent variable factor was set to 15. The results showed in Figure 3 indicating that the overall trend in the number of sample variables decreased gradually, and the trend of the curve was flattening. The cross-validated root means square error (RMSECV) curve first decreased and then increased (plot a and b in Figure 3). The RMSECV had been decreasing with the elimination of irrelevant information. However, when part of the useful information was eliminated, the RMSECV increased significantly, and the model appeared over-fitting. It was interesting that with the stability of useful information became to 0, the RMSECV correspondence would also increase (plot c in Figure 3). The modeling effect is best when the number of wavelength variable subsets was 23. Finally, 50 wavelengths were selected.

**Figure 4:**
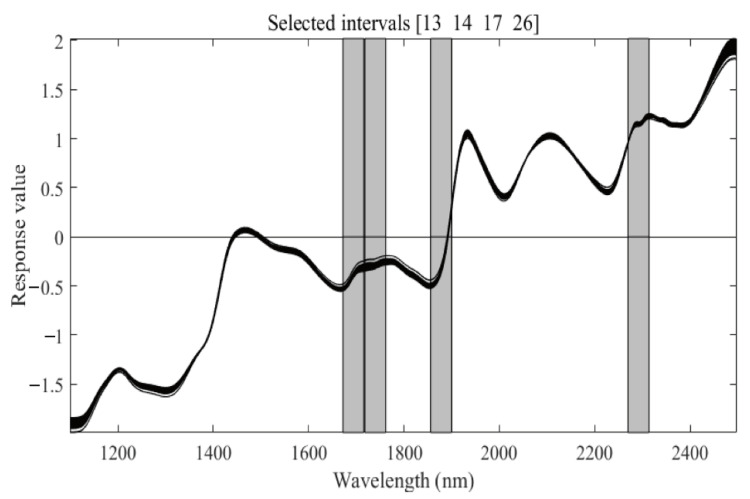
SiPLS feature selection results of mp5. Note: The black curve in the figure is the preprocessed spectrum data map, and the gray area is the characteristic band selected by the SiPLS method.

When using the SiPLS method, the spectral data were divided into 30 intervals. For each interval, 4 sub-intervals were jointly divided, and the PLS model was established. The maximum number of factors for the PLS model was 15. The final sequence numbers of the optimal feature interval were 13, 14, 17, and 26 (Figure 4). Additionally, 92 wavelengths were selected.

Predicted results of corn oil content model transferring by mp6 to mp5 under different conditions are shown in Table 1. It can be seen that the RMSECV values of the mp5 correction model are reduced to varying degrees after the optimization of the feature selection methods. However, the correction model built with the SAFS algorithm is not the best. After using different calibration transfer algorithms to predict the samples of the mp6 prediction set, it can be found that the prediction results after feature selection by the SAFS algorithm are better than others, the value of determination coefficient *R*^2^ is maintained at a high state compared to other methods, and the root mean square error of prediction (RMSEP) is the lowest. Among them, when the PDS, SST, and OSC algorithms are used for calibration transfer, and the RMSEP values of the prediction model, optimized by the SAFS algorithm, are 0.0731, 0.0893, and 0.1320. Additionally, the *R*^2^ values are 0.8145, 0.7234, and 0.5556, respectively. However, there is an exception that when using the SBC algorithm, the “none” feature selection prediction effect is better, and its RMSEP is lowest. However, the RMSEP of SAFS is lower than CARS and SiPLS, indicating that its generalization ability is still competent.

At the same time, it can be found that the prediction effect of the calibration transfer algorithm with standard samples is better than the method without standard samples. Among the algorithms with standard samples, the prediction results of the PDS and SST algorithms based on the data conversion idea are better than the SBC algorithm that only corrects the prediction results. ATLD, spectral regression, and CAWS methods were used by Liu et al. [22], Peng et al. [21] and Xu et al. [30] to transfer the same data (mp6 to mp5), respectively, and the RMSEP of the results were 0.1519, 0.098, and 0.096. Compared with these methods, the results of this study under the same preprocessing and calibration transfer algorithm were better (RMSEP = 0.0731). Therefore, the SAFS algorithm is reliable in the selection of calibration transfer features.

### 4.2. Calibration Transfer of Larch Dataset

For calibration transfers in larch wood dataset involved in different moisture content, the wavelengths of the master and slave model spectral data were selected by CARS and SiPLS methods. The parameter settings are the same as the corn data. Finally, 70 wavelengths were selected by using the CARS method. The sequence numbers of the optimal feature intervals selected by the SiPLS method are 12, 13, 16, and 18. Additionally, a total of 92 wavelengths were selected.

Quantitative prediction model of larch wood density of 70%, 50%, 30%, and 10% moisture content were constructed, respectively. Similar to the results in Table 1, The overall prediction performance of the calibration transfer model optimized by the SAFS algorithm is the best (Table 2, Table 3 and Table 4). However, there are a few exceptions. For instance, the RMSEP value without feature selection is the lowest when the OSC method is used for calibration transfer (Table 2); the RMSEP values without feature selection were maintained at more satisfactory levels when calibration transfer was performed using the SBC and SST algorithms (Table 3). However, the SAFS algorithm still obtains the optimal transfer result in the feature selection algorithms. It can be seen that the wavelengths with greater stability between the master and slave instruments data are fully considered by SAFS when selecting feature variables.

For the larch wood prediction models transferring by 70% to 10% moisture content, the prediction effect (*R*^2^ = 0.4852, RMSEP = 0.0475) is best, when the SAFS is used to select wavelengths and the SBC is used for calibration transfer. For larch wood prediction models transferring by 50% to 10% moisture content, the prediction effect (*R*^2^ = 0.3263, RMSEP = 0.0563) is best, when “none” feature selection is performed, and the SBC is used for calibration transfer. For the larch wood prediction model transferring by 30% to 10% moisture content, the prediction effect of the calibration transfer by PDS algorithm is the best (*R*^2^ = 0.5333, RMSEP = 0.0486), when the SAFS is used to select wavelengths. Therefore, the baseline drift, absorption peak drift, and partial peak shape changes of the spectrum in different detection environments can be resolved by the SAFS algorithm, thereby improving the prediction of the transfer model.

As illustrated in Table 2, Table 3 and Table 4, the minimum RMSECV in different moisture content conditions of 70%, 50%, and 30% are 0.0282, 0.0281, and 0.0279, respectively, which signifies that the experimental group whose moisture content is close to the control group has a relatively good correction model when other conditions remain unchanged; the prediction accuracy of the cross-validation correction model decreases with the increase in the moisture content.

**Figure 5:**
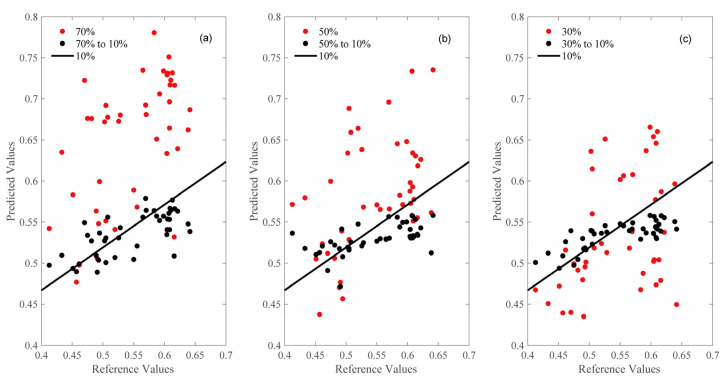
Correlation between standard test values and NIR predicted values for 70% (**a**), 50% (**b**), and 30% (**c**) moisture content, before and after calibration transfer. Note: The red points in the figure are the results directly predicted by the main model before the slave instrument is transmitted, and the black points are the results predicted by the main model after the slave instrument is transmitted. The black fitted line in the figure is the prediction result of the main model.

Based on the results depicted Figure 5 Most of the predicted and standard values of 70% and 50% moisture content are located above the 10% moisture content prediction straight line, indicating a tendency for the overall prediction to be higher. Additionally, most of the predicted and standard values of 30% moisture content are below the 10% moisture content prediction straight line, indicating a tendency for the overall prediction to be lower. Different moisture content affects the response function, large systematic errors will occur when the 10% moisture content model is used to predict spectra under other moisture content conditions. After feature selection by SAFS and calibration transfer by PDS, the corresponding points of the predicted value and the standard value are roughly distributed on both sides of the prediction line of the master instrument, which indicates that the calibration transfer optimized by the SAFS algorithm can eliminate most of the differences between the master and slave instruments effectively, and improve the prediction effect of the forecast set of the slave instrument significantly.

### 4.3. Comparison of SAFS, CARS, and SiPLS Feature Selection Results

**Figure 6:**
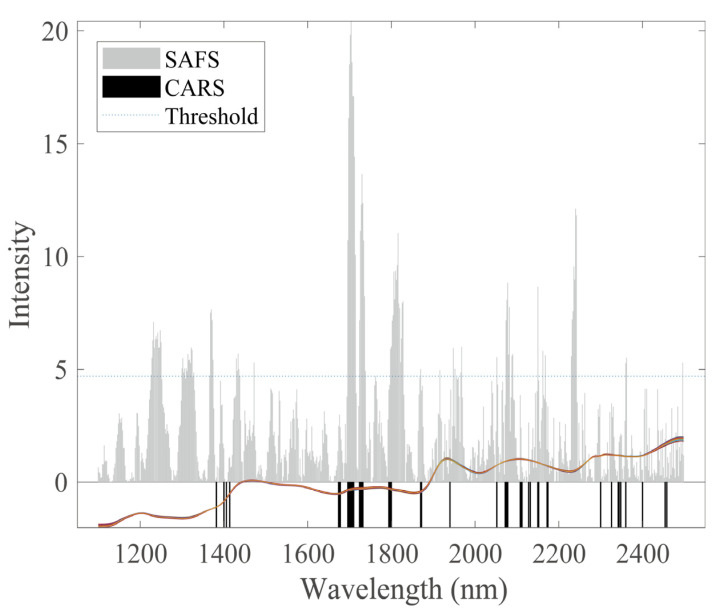
Comparison of selected wavelengths by SAFS and CARS for corn dataset. Note: The gray part in the figure represents the stability value calculated by SAFS, the horizontal dashed line represents the stability threshold T, and the black part in the figure represents the wavelengths selected by CARS, and the colored line in the figure is the mp5 spectral data curve.

To further explore the optimization principles of SAFS, the three feature selection methods of SAFS, CARS, and SiPLS in Table 1 are combined with the PDS algorithm for analysis. The RMSECV of cross-validation correction model established by CARS is the lowest in each group of experiments, which signifies that the selected wavelengths have the highest correlation with the target component. The comparison results of wavelengths selected by SAFS and CARS on the corn dataset are shown in Figure 6. Where the gray part is the stability value calculated by SAFS, the horizontal dashed line is the stability threshold T, and the part above the threshold represents the wavelengths selected by SAFS. The black part represents the wavelengths selected by CARS. It can be seen that in the 1228–1250 nm, 1300–1328 nm, and 1810–1828 nm bands, the stability calculated by SAFS is greater than the threshold, but CARS did not select them, and these bands are contained in the double-frequency absorption band of the O-H bond of water molecules [45], this may be the reason for the poor calibration transfer results of the CARS model. For the 2300–2498 nm band, the wavelengths had not been selected by the SAFS are located in the first-order, double-frequency absorption band of the O-H bond of water molecules in the long-wave near-infrared analysis. This may be the main reason why the cross-validation model built by SAFS does not perform well enough. The remaining wavelengths selected by SAFS and CARS are similar.

**Figure 7:**
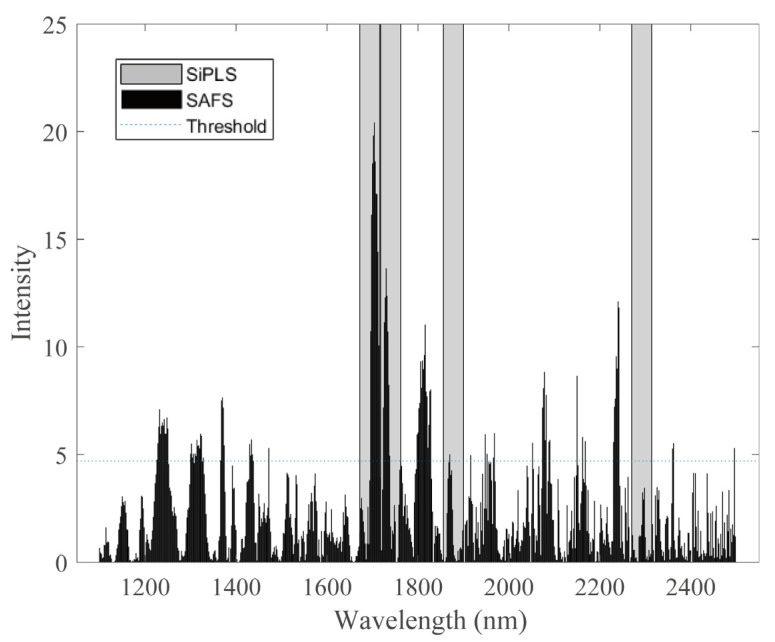
Comparison of selected wavelengths by SAFS and SiPLS for corn dataset. Note: The black part in the figure is the stability value calculated by SAFS, the horizontal dashed line is the stability threshold T, and the gray part is feature intervals selected by SiPLS.

As shown in Table 1, Table 2, Table 3 and Table 4, the RMSECV value of the model built by the SiPLS method is slightly smaller than those of the SAFS algorithm, but it yields the worst RMSEP values of all the methods. For the corn dataset with the 1228–1250 nm, 1300–1328 nm, and 1800–1828 nm band, the wavelengths selected by SiPLS is obviously missing in these parts (Figure 7). In the 1672–1762 nm and 1856–1900 nm bands, many wavelengths whose stability are far below the threshold are eliminated by SAFS, but selected by SiPLS. This may be the reason why the SiPLS model obtains the worst calibration transfer results. In the 2270–2498 nm band, similar to the CARS results, the wavelengths had been not selected by SAFS. SAFS is prone to lack of selection in the long-wave near-infrared spectroscopy, which may be caused by interference such as noise, so it can be optimized by replacing the pretreatment method or combining with the traditional feature selection method.

According to the above results, for both datasets, the models with the best calibration performance performs poorly during transmission, but the transfer model optimized by the SAFS algorithm can consistently produce the best prediction results, even though its calibration performance is not optimal. Compared with CARS and SiPLS methods, the wavelengths selected by the SAFS have more representative, which means that SAFS can eliminate the variables of poor consistency and stability between master and slave instruments better (Figure 6 and Figure 7). Specifically, in the calibration transfer process, the consistency and stability between variables play a more important role than weights.

## 5. Conclusions

In the process of calibration transfer, appropriate consistent feature selection can reduce the variable redundancy and improve the prediction effect of the model. Therefore, a new feature selection method is proposed, called SAFS. It is used to optimize the common wavelengths of the master and slave instruments before constructing the transfer model, reduce the data dimension, and improve the prediction and efficiency of the traditional calibration transfer algorithm. Specifically, the Monte Carlo sampling method is used to calculate the absolute value of the correlation stability of the spectral data between master and slave instruments, and the genetic algorithm is used to find the optimal threshold of transfer effect (the maximum fitness function value of genetic algorithm) to eliminate invalid information variables whose stability is lower.

Two datasets of corn and larch wood were used to verify the reliability of the method. The calibration transfer was achieved through four classical methods with different principles, and a comparative analysis was also made with other feature selection methods. The results were shown that the overall prediction performance of the transfer model established by using the characteristic wavelength selected by SAFS was the best. SAFS was an algorithm specially developed for model transfer. The model performance optimized by SAFS and traditional feature selection methods were compared and analyzed. Although the correction performance of the SAFS optimized model was not optimal, its transfer performance was still much better than the traditional method. SAFS can be used as a framework algorithm that can be further optimized by changing the preprocessing method or by combining it with traditional feature selection methods.

In summary, the SAFS algorithm has been verified in both ideal state samples (corn dataset) and actual complex samples (larch wood dataset), and its simple principle and operability can improve the accuracy and efficiency of calibration transfer, which has practical application value. In practice, there are still many factors affecting wood density, and we will further investigate the optimized performance of SAFS under the influence of multiple factors. Additionally, the experimental results of SAFS algorithm on laboratory NIR spectrometers (mp5, mp6) and portable NIR spectrometers (LabSpec) have achieved satisfactory results. The improvement of SAFS algorithm for small NIR spectrometers will be one of the next research focuses.

## Figures and Tables

**Table 1:** Predicted results of corn oil content model transferring by mp6–mp5 under different conditions.

Calibration Transfer	Variable Selection	Spectral Variable	RMSECV	*R* ^2^	RMSEP	Latent Variable	Threshold Number
SBC	none	700	0.1156	**0.6953**	**0.1006**	7	/
	SAFS	78	0.0931	0.6196	0.1096	8	5.3659
	CARS	50	0.0656	0.6173	0.1122	13	/
	SiPLS	92	0.0675	0.6113	0.1148	14	/
PDS	none	700	0.1129	0.7027	0.0926	7	/
	SAFS	99	0.0909	**0.8145**	**0.0731**	12	4.6952
	CARS	50	0.0660	0.7701	0.0852	13	/
	SiPLS	92	0.0660	0.5260	0.1169	14	/
SST	none	700	0.1139	0.7226	0.0894	7	/
	SAFS	96	0.1018	**0.7234**	**0.0893**	8	4.8376
	CARS	50	0.0700	0.6655	0.0982	13	/
	SiPLS	92	0.0704	0.3575	0.1361	14	/
OSC	none	700	0.1175	0.4113	0.1435	7	/
	SAFS	116	0.1017	**0.5556**	**0.1320**	13	2.1608
	CARS	50	0.0624	0.3136	0.1406	13	/
	SiPLS	92	0.0687	0.4554	0.1353	14	/

Note: The highest *R*^2^ and lowest RMSEP values for each category are shown in bold black.

**Table 2:** Predicted results of larch wood density model transferring by 70% to 10% moisture content under different conditions.

Method	Variable Selection	Spectral Variables	RMSECV	*R* ^2^	RMSEP	Latent Variable	Threshold Number
SBC	none	2151	0.0435	0.3574	0.0525	7	/
	SAFS	449	0.0372	**0.4852**	**0.0475**	5	3.8110
	CARS	70	0.0285	0.4343	0.0488	7	/
	SiPLS	288	0.0371	0.3307	0.0659	7	/
PDS	none	2151	0.0446	0.2876	0.0532	7	/
	SAFS	255	0.0363	**0.3580**	**0.0505**	5	5.0046
	CARS	70	0.0291	0.3349	0.0513	6	/
	SiPLS	288	0.0387	0.0226	0.0669	5	/
SST	none	2151	0.0438	0.2978	0.0528	8	/
	SAFS	237	0.0346	**0.3301**	**0.0515**	5	5.2223
	CARS	70	**0.0282**	0.0534	0.0692	7	/
	SiPLS	288	0.0375	0.3034	0.0527	7	/
OSC	none	2151	0.0464	**0.4582**	**0.0639**	8	/
	SAFS	170	0.0344	0.3999	0.0862	5	6.5015
	CARS	70	0.0286	0.0164	0.0974	6	/
	SiPLS	288	0.0371	0.0996	0.1676	5	/

Note: The highest *R*^2^ and lowest RMSEP values for each category are shown in bold black. The lowest RMSECV values for each moisture content are shown in bold red.

**Table 3:** Predicted results of larch wood density model transferring by 50%–10% moisture content under different conditions.

Method	Variable Selection	Spectral Variable	RMSECV	*R* ^2^	RMSEP	Latent Variable	Threshold Number
SBC	none	2151	0.0468	**0.3263**	**0.0563**	7	/
	SAFS	255	0.0386	0.2502	0.0590	7	9.6139
	CARS	70	0.0289	0.1303	0.0681	6	/
	SiPLS	288	0.0383	0.0359	0.0713	5	/
PDS	none	2151	0.0446	0.0753	0.0606	8	/
	SAFS	40	0.0374	**0.1707**	**0.0573**	7	4.9920
	CARS	70	0.0286	0.0763	0.0605	6	/
	SiPLS	288	0.0377	0.1693	0.0574	5	/
SST	none	2151	0.0469	**0.2758**	**0.0565**	7	/
	SAFS	18	0.0388	0.2663	0.0613	12	8.4456
	CARS	70	0.0289	0.0048	0.0628	7	/
	SiPLS	288	0.0369	0.0035	0.0780	5	/
OSC	none	2151	0.0462	0.1014	0.0745	5	/
	SAFS	30	0.0354	**0.2343**	**0.0714**	9	8.8910
	CARS	70	**0.0281**	0.0582	0.1189	6	/
	SiPLS	288	0.0384	0.0260	0.1776	5	/

Note: The highest *R*^2^ and lowest RMSEP values for each category are shown in bold black. The lowest RMSECV values for each moisture content are shown in bold red.

**Table 4:** Predicted results of larch wood density model transferring by 30%–10% moisture content under different conditions.

Method	Variable Selection	Spectral Variable	RMSECV	*R* ^2^	RMSEP	Latent Variable	Threshold Number
SBC	none	2151	0.0467	0.3133	0.0619	7	/
	SAFS	784	0.0377	0.3204	0.0575	8	2.3423
	CARS	70	0.0294	0.0632	0.0689	6	/
	SiPLS	288	0.0675	**0.6113**	**0.1148**	14	/
PDS	none	2151	0.0465	0.2027	0.0562	7	/
	SAFS	368	0.0340	**0.5333**	**0.0486**	7	4.3645
	CARS	70	**0.0279**	0.5154	0.0438	6	/
	SiPLS	288	0.0356	0.3046	0.0525	7	/
SST	none	2151	0.0459	0.0152	0.0625	7	/
	SAFS	214	0.0372	**0.4131**	**0.0495**	7	5.3268
	CARS	70	0.0287	0.4001	0.0505	6	/
	SiPLS	288	0.0377	0.2973	0.0528	7	/
OSC	none	2151	0.0469	0.0132	0.0773	7	/
	SAFS	72	0.0364	**0.0995**	**0.0752**	7	11.7870
	CARS	70	0.0289	0.0301	0.1377	6	/
	SiPLS	288	0.0381	0.0455	0.2116	6	/

Note: The highest *R*^2^ and lowest RMSEP values for each category are shown in bold black. The lowest RMSECV values for each moisture content are shown in bold red.

## Data Availability

The data presented in this study are available on request from the corresponding author. The data are not publicly available due to it is being used to apply for project.

## References

[1] Schimleck L., Matos J.L.M., Higa A., Trianoski R., Prata J.G., Dahlen J. (2020). Classifying wood properties of loblolly pine grown in Southern Brazil using NIR-Hyperspectral imaging. Forests.

[2] Caramês E.T.D.S., Piacentini K.C., Alves L.T., Pallone J.A.L., Rocha L.D.O. (2020). NIR spectroscopy and chemometric tools to identify high content of deoxynivalenol in barley. Food Addit. Contam. Part A.

[3] Biagi D., Nencioni P., Valleri M., Calamassi N., Mura P. (2021). Development of a Near Infrared Spectroscopy method for the in-line quantitative bilastine drug determination during pharmaceutical powders blending. J. Pharm. Biomed. Anal..

[4] He K., Zhong M., Li Z., Liu J. (2020). Near-infrared spectroscopy for the concurrent quality prediction and status monitoring of gasoline blending. Control. Eng. Pract..

[5] Feudale R.N., Woody N.A., Tan H., Myles A.J., Brown S.D., Ferré J. (2002). Transfer of multivariate calibration models: A review. Chemom. Intellig. Lab. Syst..

[6] Fan S.X., Li J.B., Xia Y., Tian X., Guo Z.M., Huang W.Q. (2019). Long-term evaluation of soluble solids content of apples with biological variability by using near-infrared spectroscopy and calibration transfer method. Postharvest Biol. Technol..

[7] Li X.Y., Ren G.X., Fan P.P., Liu Y., Sun Z.L., Hou G.L., Lv M.R. (2020). Study on the Calibration Transfer of Soil Nutrient Concentration from the Hyperspectral Camera to the Normal Spectrometer. J. Spectrosc..

[8] Shi Y.-Y., Li J.-Y., Chu X.-L. (2019). Progress and Applications of Multivariate Calibration Model Transfer Methods. Chin. J. Anal. Chem..

[9] Beć K.B., Grabska J., Huck C.W. (2021). Principles and Applications of Miniaturized Near-Infrared (NIR) Spectrometers. Chem.-A Eur. J..

[10] Hoffmann U., Pfeifer F., Hsuing C., Siesler H.W. (2016). Spectra Transfer Between a Fourier Transform Near-Infrared Laboratory and a Miniaturized Handheld Near-Infrared Spectrometer. Appl. Spectrosc..

[11] Zamora-Rojas E., Perez-Marin D., De Pedro-Sanz E., Guerrero-Ginel J.E., Garrido-Varo A. (2012). Handheld NIRS analysis for routine meat quality control: Database transfer from at-line instruments. Chemom. Intellig. Lab. Syst..

[12] van Kollenburg G., Weesepoel Y., Parastar H., van den Doel A., Buydens L., Jansen J. (2020). Dataset of the application of handheld NIR and machine learning for chicken fillet authenticity study. Data Brief.

[13] Woody N.A., Feudale R.N., Myles A.J., Brown S.D. (2004). Transfer of Multivariate Calibrations between Four Near-Infrared Spectrometers Using Orthogonal Signal Correction. Anal. Chem..

[14] Blank T.B., Sum S.T., Brown S.D., Monfre S.L. (1996). Transfer of Near-Infrared Multivariate Calibrations without Standards. Anal. Chem..

[15] Behrens A. (1997). Multivariate calibration standardization used for the reduction of standards needed for multivariate calibration in inductively coupled plasma mass spectrometry. Spectrochim. Acta Part B.

[16] Wang Y., Veltkamp D.J., Kowalski B.R. (1991). Multivariate Instrument Standardization. Anal. Chem..

[17] Bouveresse E., Massart D.L. (1996). Standardisation of near-infrared spectrometric instruments: A review. Vib. Spectrosc..

[18] Bouveresse E., Massart D.L., Dardenne P. (1994). Calibration transfer across near-infrared spectrometric instruments using Shenk’s algorithm: Effects of different standardisation samples. Anal. Chim. Acta.

[19] Du W., Chen Z.P., Zhong L.J., Wang S.X., Yu R.Q., Nordon A., Littlejohn D., Holden M. (2011). Maintaining the predictive abilities of multivariate calibration models by spectral space transformation. Anal. Chim. Acta.

[20] Fan W., Liang Y., Yuan D., Wang J. (2008). Calibration model transfer for near-infrared spectra based on canonical correlation analysis. Anal. Chim. Acta.

[21] Peng J., Peng S., Jiang A., Tan J. (2011). Near-infrared calibration transfer based on spectral regression. Spectrochim. Acta Part A.

[22] Liu Y., Cai W., Shao X. (2014). Standardization of near infrared spectra measured on multi-instrument. Anal. Chim. Acta.

[23] Liu Y., Cai W., Shao X. (2016). Linear model correction: A method for transferring a near-infrared multivariate calibration model without standard samples. Spectrochim. Acta Part A.

[24] Yu Y., Huang J.P., Liu S.S., Zhu J., Liang S.L. (2021). Cross target attributes and sample types quantitative analysis modeling of near-infrared spectroscopy based on instance transfer learning. Measurement.

[25] Li X., Li Z., Yang X., He Y. (2021). Boosting the generalization ability of Vis-NIR-spectroscopy-based regression models through dimension reduction and transfer learning. Comput. Electron. Agric..

[26] Shan P., Zhao Y., Wang Q., Wang S., Ying Y., Peng S. (2021). A nonlinear calibration transfer method based on joint kernel subspace. Chemom. Intellig. Lab. Syst..

[27] Ni L., Chen H., Hong S., Zhang L., Luan S. (2021). Near infrared spectral calibration model transfer without standards by screening spectral points with scale invariant feature transform from master samples spectra. Spectrochim. Acta, Part A.

[28] Zheng K.Y., Feng Y.H., Zhang W., Huang X.W., Li Z.H., Zhang D., Shi J.Y., Zou X.B. (2021). Iterative Interval Backward Selection Algorithm and Its Application in Calibration Transfer of Near Infrared Spectra. Spectrosc. Spect. Anal..

[29] Zhang X.Y., Li Q.B., Zhang G.J. (2014). Calibration transfer without standards for spectral analysis based on stability competitive adaptive reweighted sampling. Spectrosc. Spect. Anal..

[30] Xu Z., Fan S., Cheng W., Liu J., Zhang P., Yang Y., Xu C., Liu B., Liu J., Wang Q. (2020). A correlation-analysis-based wavelength selection method for calibration transfer. Spectrochim. Acta Part A.

[31] Li L.S., Jang X.G., Li B., Liu Y.D. (2021). Wavelength selection method for near-infrared spectroscopy based on standard-sample calibration transfer of mango and apple. Comput. Electron. Agric..

[32] Zhang L., Li Y., Huang W., Ni L., Ge J. (2020). The method of calibration model transfer by optimizing wavelength combinations based on consistent and stable spectral signals. Spectrochim. Acta Part A.

[33] Zheng K., Li Q., Wang J., Geng J., Cao P., Sui T., Wang X., Du Y. (2012). Stability competitive adaptive reweighted sampling (SCARS) and its applications to multivariate calibration of NIR spectra. Chemom. Intellig. Lab. Syst..

[34] Jiang H., Wang J., Chen Q. (2021). Comparison of wavelength selected methods for improving of prediction performance of PLS model to determine aflatoxin B1 (AFB1) in wheat samples during storage. Microchem. J..

[35] Han Q.J., Wu H.L., Cai C.B., Xu L., Yu R.Q. (2008). An ensemble of Monte Carlo uninformative variable elimination for wavelength selection. Anal. Chim. Acta.

[36] Ho T.X., Schimleck L.R., Sinha A. (2021). Utilization of genetic algorithms to optimize Eucalyptus globulus pulp yield models based on NIR spectra. Wood Sci. Technol..

[37] Dahali R., Md. Tahir P., Roseley A.S.M., Hua L.S., Bakar E.S., Ashaari Z., Abdul Rauf M.R., Zainuddin N.A., Mansoor N.S. (2021). Influence of Chrysoporthe deuterocubensis Canker Disease on the Physical and Mechanical Properties of Eucalyptus urograndis. Forests.

[38] Li Y., Pan T., Li H., Chen S. (2020). Non-invasive quality analysis of thawed tuna using near infrared spectroscopy with baseline correction. J. Food Process Eng..

[39] Xu M.X., Chu X.Y., Fu Y.S., Wang C.J., Wu S.H. (2021). Improving the accuracy of soil organic carbon content prediction based on visible and near-infrared spectroscopy and machine learning. Environ. Earth Sci..

[40] Tiecher T., Moura-Bueno J.M., Caner L., Minella J.P.G., Evrard O., Ramon R., Naibo G., Barros C.A.P., Silva Y.J.A.B., Amorim F.F. (2021). Improving the quantification of sediment source contributions using different mathematical models and spectral preprocessing techniques for individual or combined spectra of ultraviolet–visible, near- and middle-infrared spectroscopy. Geoderma.

[41] Xie Y., Zhou C., Tu C., Zhang Z.L., Wang J.F. (2017). Quantitative Determination of Ferulic Acid Content in Chrysanthemum Morifolium cv. (Chuju) Continuous Cropping Soil Using Near Infrared Spectroscopy. Chin. J. Anal. Chem..

[42] Peng Y.F., Luo H.P., Luo X.N., Zhan Y. (2014). SPXY sample classification method and successive projections algorithm combined with near-infrared spectroscopy for the determination of total sugar content of southern xinjiang jujube. Adv Mat Res.

[43] Jiang W., Lu C., Zhang Y., Ju W., Wang J., Xiao M. (2019). Molecular spectroscopic wavelength selection using combined interval partial least squares and correlation coefficient optimization. Anal. Methods.

[44] Li M., Han D., Liu W. (2019). Non-destructive measurement of soluble solids content of three melon cultivars using portable visible/near infrared spectroscopy. Biosys. Eng..

[45] Wood J., Turner P.H. (2003). Monitoring of Itaconic Acid Hydrogenation in a Trickle Bed Reactor Using Fiber-Optic Coupled Near-Infrared Spectroscopy. Appl. Spectrosc..

